# Dry Heat as a Potential Decontamination Method on the Filtration Efficiency of Filtering Facepiece Respirators

**DOI:** 10.3390/ijerph19127167

**Published:** 2022-06-11

**Authors:** Zhixu Jin, Chenchen Sun, Wending Wu, Xiaobing Yang

**Affiliations:** 1 School of Engineering and Technology, China University of Geosciences (Beijing), Beijing 100083, China; 1002194108@cugb.edu.cn (Z.J.); wdwu.e@outlook.com (W.W.); 2 State Key Laboratory of NBC Protection for Civilian, Beijing 100191, China; 3 Beijing Key Laboratory of Ambient Particles Health Effects and Prevention Techniques, Beijing 100191, China

**Keywords:** filtering facepiece respirators, heat treatment, filtration efficiency, neural network

## Abstract

Filtering facepiece respirators have been widely used in the fields of occupational health and public hygiene, especially during the COVID-19 pandemic. In particular, disposable respirators have been in high demand, and the waste generated from these disposable products poses a problem for the environment. Here, we aimed to test a practical decontamination method to allow for the reuse of KN95 respirators. In this study, three types of KN95 respirators were heated at 80 °C and 90 °C for different durations (15 min, 30 min, 45 min, 1 h, 2 h, 3 h, 4 h, 6 h, 8 h, 10 h, and 24 h). The filtration efficiencies of the tested KN95 respirators before and after heating were measured, and the changes in microstructure were imaged with a scanning electron microscope (SEM). In addition, a neural network model based on the nonlinear autoregressive with external input (NARX) to predict the filtration efficiency of the KN95 respirator was established. The results show that the temperature and time of dry heating affected particle prevention. The higher the temperature and the longer the heating time, the more obvious the decline in the filtration efficiency of the respirators. When the heating temperature reached 100 °C, the respirator may be no longer suitable for reuse. These results show that a dry heat temperature between 70 °C and 90 °C, and a heating time between 30 min and 2 h is assumed to be a suitable and effective decontamination method for respirators.

## 1. Introduction

In China, there were 14,367 new cases of occupational pneumoconiosis in 2020, and 6668 cases resulted in death [1]. Studies have shown that a filtering facepiece respirator can effectively filter particles such as smoke, dust, haze, and microorganisms in the air, thus reducing the deposition of particles in human alveoli and effectively preventing diseases [2,3,4]. In the same year, the COVID-19 virus spread all over the world, and many studies confirmed the effectiveness of filtering facepiece respirators to protect against virus particles and reduce the spread of the virus [5,6,7,8,9,10,11]. In the China National Standard GB 2626-2019 [12], according to filtration performance, the filters of respirators are categorized as either KN or KP. KN is suitable for filtering non-oil particles, while KP is suitable for filtering oil-based particles. According to the filtration level, respirators are divided into three grades: 90, 95, and 100, corresponding to filter efficiency equal to or greater than 90%, 95%, and 99.97%, respectively.

At present, the global epidemic is still in a recurrent stage, and the possibility of a shortage of respirators remains problematic [13]. In the next few years, we may anticipate the global need for filtering facepiece respirators and a high consumption of respirators. Under natural conditions, respirators are resistant to degradation over short periods; thus, used respirators become hazardous waste, which poses a serious threat to biological health [14,15]. Therefore, effective and feasible disinfection methods of respirators to improve their reusability are urgently needed.

To date, the disinfection methods of respirators mainly include dry heat [16,17,18,19,20,21,22], water vapor [16,17,23,24,25], chemical disinfectant [17,24], ultraviolet rays [16,17,18,23,24,26], hydrogen peroxide vapor [17,18,20,23,26,27,28,29], microwave radiation [17,18,19], supercritical CO_2_ [29], high pressure steam [17,18], gamma radiations [30,31], and ethylene oxide [17,26]. However, limitations of these methods persist. For example, ultraviolet germicidal irradiation (UVGI) may only be effective if it makes direct contact with the surface. Considering this and the fact that N95 respirators have a curvature, disinfection with UVGI alone may result in incomplete disinfection [32,33]. Ethylene oxide is not recommended because of the potential carcinogenicity and flammability [17]. Gamma radiation is not recommended because of a significant drop in filtration efficiency [30,31]. Chemical disinfectant is not recommended because of its strong smell and structural damage to the filter material [17,33,34]. In contrast, dry heat, hydrogen peroxide vapor, and supercritical CO_2_ have been proven as effective methods for decontamination [17,28,29,33,35,36,37,38,39]. The dry heat technique is of particular interest because of its simple operation and the fact that there is no use and release of chemicals. The China pneumonia diagnosis and treatment program for novel coronavirus infection (Trial Version 5) clearly states that heating at 56 °C for 30 min can effectively inactivate coronavirus [40]. There are studies in the literature to prove that the inactivation efficiency of bacteria and viruses increases as the temperature rises [41,42,43]. Therefore, it is necessary to optimize the temperature and heating duration. Pascoe et al. confirmed a significant decrease in viral particle activity at 70 °C [19]. Liao et al. determined that dry heating at 100 °C did not affect the filtration efficiency of the respirator [24]. Mackenzie et al. found a decrease in the filter efficiency of the respirator at a dry heating temperature of 125 °C [16], Yim, Sales, and Viscusi reported the degradation of the filter microstructure when the temperature was increased to 160 °C [22,44,45]. Therefore, dry heat treatment in the temperature range of 70 °C–100 °C can inactivate potential viruses, while ensuring the filtration efficiency of the respirator [19,24]. In our study, we measured the changes in filtration efficiency and filter microstructure upon dry heat treatment at 80–90 °C, which fills the experimental gap in the current studies. In addition, a neural network model based on the nonlinear autoregressive with external inputs (NARX) was used to establish a prediction model of respirator filtration efficiency, and the prediction of the change in efficiency was successfully realized. This research is of great significance in enhancing the reuse of filtering facepiece respirators, alleviating product shortages, and reducing the environmental pollution.

## 2. Materials and Methods

### 2.1. Filtering Facepiece Respirator Description

**Figure 1:**
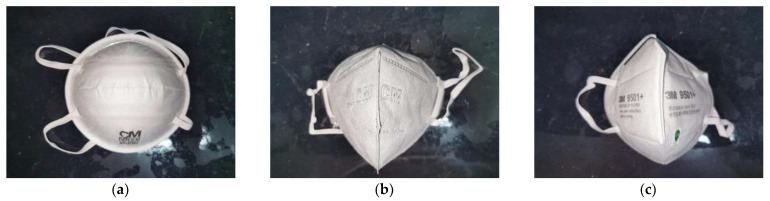
The three typical KN95 disposable facepiece respirators: (**a**) CM 8228-1 cup-shaped KN95, (**b**) CM 6002A head-mounted KN95, (**c**) 3M 9501+ folding KN95.

In this study, three typical KN95 disposable facepieces were selected, namely, a CM (Chaomei) 8228-1 cup-shaped KN95 respirator; a CM (Chaomei) 6002A head-mounted KN95 respirator; and a 3M 9501+ folding KN95 respirator, as shown in Figure 1. Three typical respirators in the Chinese market were selected, which are widely used in practice. The filters of the three KN95 respirators are made of polypropylene, and the CM 6002A KN95 respirator contains an active carbon filter. All of the tested respirators used in this study have passed the China national standard GB 2626-2019, which includes temperature conditioning as a pretreatment process. All samples were sealed in the original packaging before testing.

### 2.2. Heat Pretreatment

**Figure 2:**
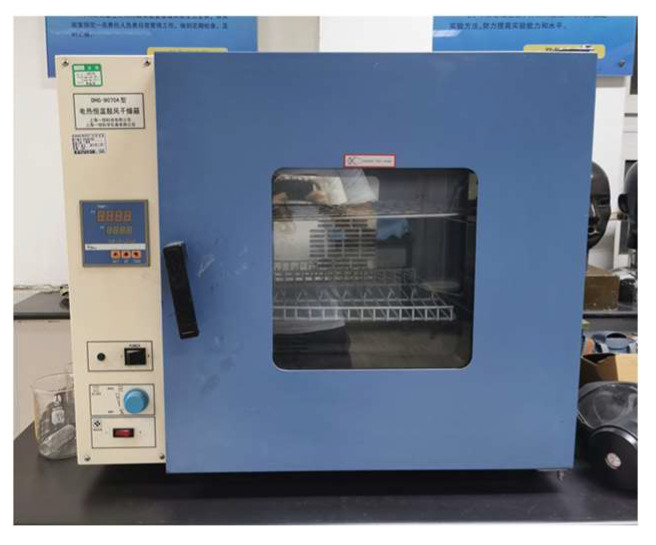
The DHG-9070A drying oven.

**Figure 3:**
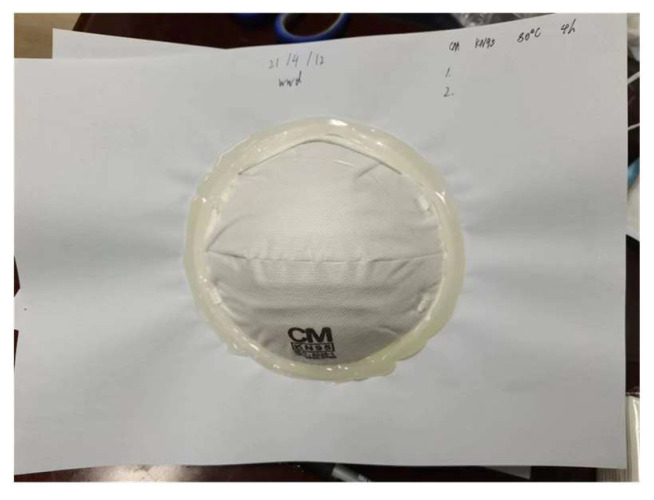
The sample after treatment.

In this experiment, the heating temperatures were 80 °C and 90 °C, and the heated durations were 15 min, 30 min, 45 min, 1 h, 2 h, 3 h, 4 h, 6 h, 8 h, 10 h, and 24 h, respectively. A DHG-9070A drying oven (Model 9070A, Shanghai Yiheng Inc., Shanghai, China) with a timing function was selected (Figure 2). Three respirators of each model were taken, and each experiment was repeated twice. The filtration efficiency of the respirators was tested at room temperature (25 °C) and the heating temperatures (80 °C and 90 °C) after each heating duration. After dry heat treatment, the respirator earrings were removed. Air holes were cut out of A4 paper. Each respirator was fixed to a sheet of A4 paper using a hot-melt adhesive to seal the edges of the respirator. The temperature, heating duration, respirator model, and testing time were marked on the A4 paper (Figure 3).

### 2.3. Filtration Efficiency Measurement

A TSI-8130 automatic filter material detector (Model 8130, TSI Inc., Shoreview, MN, USA) was selected for the experiment. Dioctyl phthalate (DOP) particles and NaCl particles were selected as the tested particles. Measurements were performed according to the method in GB 2626-2019 [12]. The particle testing parameters are shown in Table 1.

**Figure 4:**
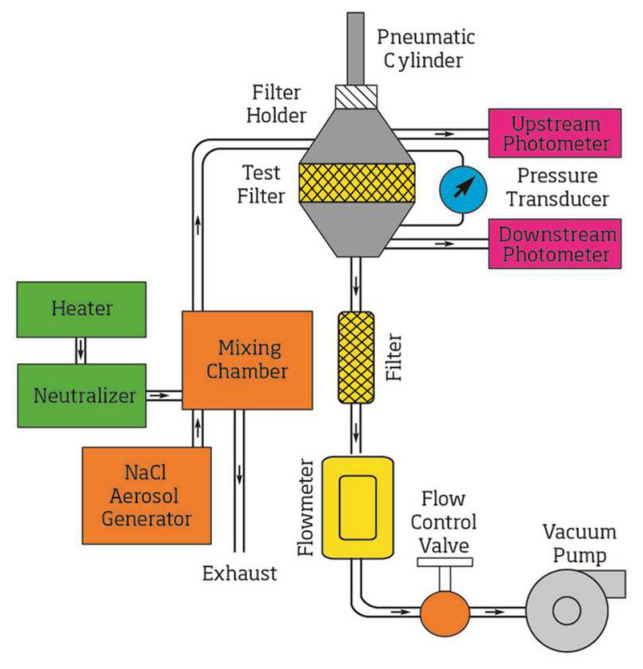
The schematic of the measuring principle of the TSI 8130 detector. Reprinted with per-mission from Ref. [46]. 2021, TSI Incorporated.

The filtration efficiency of the DOP particles was measured first, and then the filtration efficiency of the NaCl particles was measured using the same samples. The schematic description of the measuring principle of TSI 8130 is shown in Figure 4.

The particle generator of the detector produces particles. Air, in which the original particles and static electricity are removed, is mixed with the particles from the particle generator. The flow rate of the gas mixture was 85 L/min. The concentration of particles in the airflow was measured before and after they passed through the respirator. The ratio of particle concentration before and after the particles passed through the test respirator was calculated, and the filtration efficiency of the measured respirator was determined as follows:

(1) $$\eta =\left(1-\frac{C_{N,\;after}}{C_{N,\;before}}\right)\times 100\%$$

where $\;C_{N,\;before}$ is the concentration of particles before passing through the test respirator, and $C_{N,\;after}$ is the concentration of particles after passing through the test respirator.

### 2.4. Microstructure of the Respirator Filter

To observe the microstructure changes of the respirator filter after dry heat treatment, a COXEM EM-30 electron microscope (Model EM-30, COXEM Inc., Daejeon, Korea) was used to image the samples after the experiment. The scanning electron microscope (SEM) images of the respirator filter were taken at room temperature for 0 h, 80 °C for 24 h, and 90 °C for 24 h.

### 2.5. Experimental Data Processing

#### 2.5.1. Filtration Efficiency

**Figure 5:**
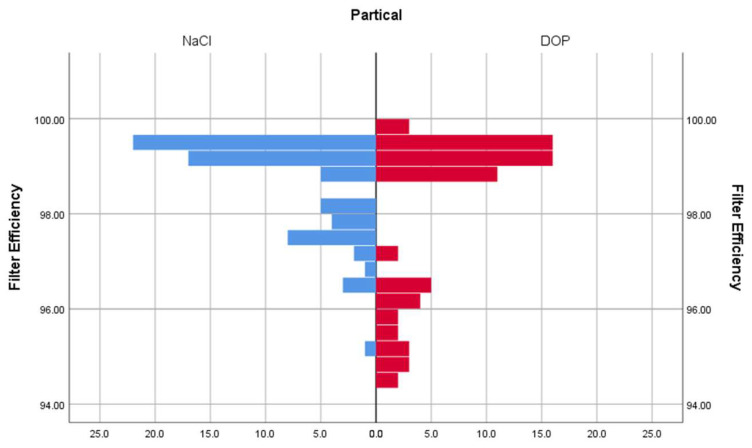
The bidirectional comparison histogram of the filtration efficiency of respirators treated with NaCl particles (blue) and DOP particles (red).

The filtration efficiency of the three samples of each model of the respirator was measured twice. After the experiment, 810 results were obtained. The DOP particle dataset consisted of 414 data points, and the NaCl particle dataset consisted of 396 data points. In the NaCl particle dataset, there were 18 data points less because one respirator of each model was used for the SEM images, and the average filtration efficiency of the two other respirators of the same model supplemented the relevant data. After eliminating gross errors, SPSS (IBM Inc., Chicago, IL, USA) was used to analyze the normal distribution of the experimental data. Through the K–S test of the data, the significance of NaCl and DOP were both less than 0.05, indicating that the two sample datasets did not obey the normal distribution. Next, we used the Mann–Whitney U test to verify whether the filtration efficiency was affected by the type of particle. To satisfy the hypothesis of the *U* test, we constructed a bidirectional comparison histogram, and the approximate distribution of the two groups of samples is shown in Figure 5. The test results *p* = 0.189 > 0.05 show that the DOP particles or NaCl particles will not affect the change in filtration efficiency under dry heat treatment.

#### 2.5.2. Microstructure

To better visualize the microstructure changes, the SEM images of the respirator filters were selected for contrast coloring. The area of the filter with relatively serious melting and agglomeration was marked with red, and the rest was marked with blue to increase the contrast. At the same time, we counted the fiber diameters in the SEM images and made histograms of the fiber diameter frequency distribution.

#### 2.5.3. Establishment of a Neural Network Model

To observe the change in filtration efficiency under the simultaneous action of time and temperature, we constructed a NARX neural network model based on the current data and aimed to predict the change in filtration efficiency. NARX is a model for nonlinear discrete systems as a nonlinear autoregressive neural network with external input. Its theoretical equation is [47,48]:

(2) $$T(t)=f\{x(t-D_{x}),x(t-D_{x-1}^{}),\;\ldots ,\;x(t-1),x(t),T(t-D_{T}),\ldots ,T(t-1)\}$$

where *T(t)* is the input; *x(t)* is the output; *D_x_* is the maximum input delay; *D_T_* is the maximum output delay; and *f* is the function.

**Figure 6:**
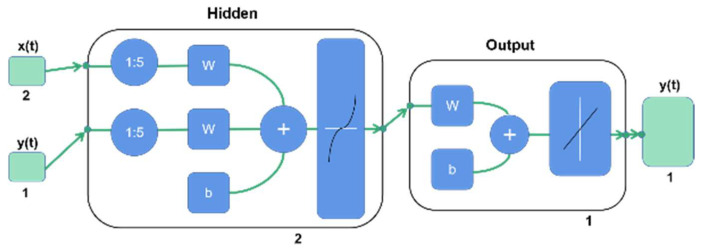
The open-loop structure of the NARX network with external input y(t).

**Figure 7:**
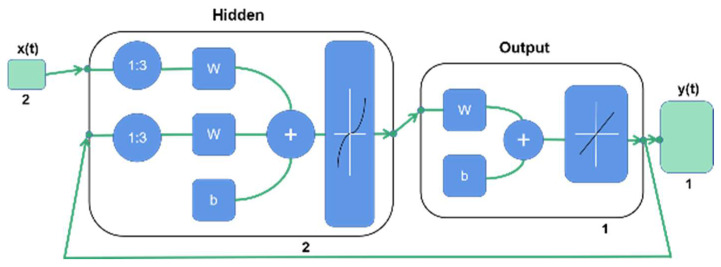
The closed-loop structure of the NARX network with no external input y(t).

After calculating the average value of repeated experimental results under the same dependent variable conditions, 138 processed data were obtained. Matlab (MathWorks. Inc., Natick, MA, USA) was used to establish the neural network model. According to the proportions of 70%, 15%, and 15%, the data were divided into training, verification, and test groups. The random partition method was used to ensure the rationality of data partition and ensure that there was no data overlap and intersection between datasets. To ensure a good training effect, the input and output delay were set to 5, which indicates that the experimental data in the input layer will be delayed by 5 sample data points based on time series. The number of neurons in the hidden layer was 2, and the number of neurons in the output layer was 1. The NARX network uses an open-loop structure (Figure 6) for network training. After the ideal training results were obtained, the network was transformed into a parallel closed-loop structure (Figure 7) by a closed-loop function for prediction. The filtering efficiency was predicted by the sim function with a trained closed-loop structure neural network.

## 3. Results

### 3.1. Filtration Efficiency

**Figure 8:**
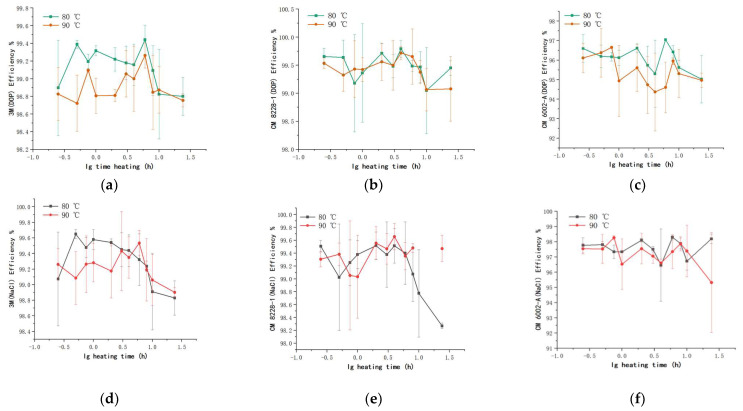
The filtration efficiency of DOP particles through (**a**) 3M KN95, (**b**) CM 8228-1KN95, and (**c**) CM 6002A respirators. The filtration efficiency of NaCl particles through (**d**) 3M KN95, (**e**) CM 8228-1KN95, and (**f**) CM 6002A respirators.

The change in filtration efficiency upon heat treatment at different temperatures and durations is shown in Figure 8. Each point represents the average of six replicate measurements, and the error bar represents the standard deviation of six data points.

The filtration efficiency of the KN95 respirator specified in GB 2626-2019 [12] was used as the reference standard. When the filtration efficiency was lower than 95%, the respirator was considered no longer usable. From Figure 8, the filtration efficiency of three KN95 disposable respirators decreased with time, whether under the action of DOP particles or NaCl particles. After heating at 80 °C or 90 °C for 24 h, the filtration efficiency of 3M KN95 and CM 8228-1 KN9 remained at 98% or greater, which met the standard and indicated continuous use. The filtration efficiency of CM 6002A KN95 before dry heat treatment was 97.1%, which was lower than that of the two types of respirators before treatment. During the dry heat treatment process at 80 °C for 10 h, the filtration efficiency of this type of respirator remained above 95%. However, at 90 °C and heating for more than 2 h, the filtration efficiency (DOP) of the CM 6002A KN95 decreased to 94.74% and then continued to decrease. The results show that the filter efficiency of the 3M KN95 and CM 8228-1 KN95 was close than that of the CM 6002A KN95.

**Figure 9:**
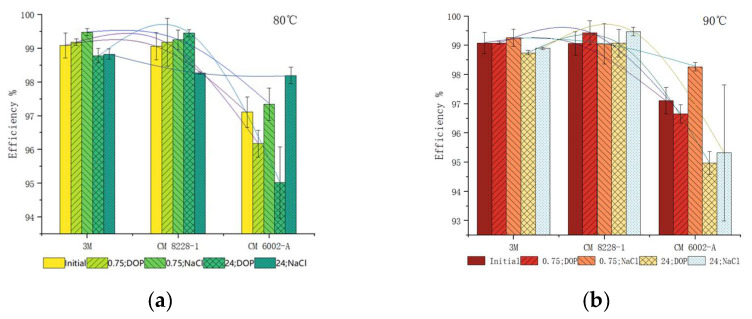
(**a**) The DOP particles and NaCl particles filtration efficiency at 80 °C for 0. 75 h and 24 h, respectively. (**b**) The DOP and NaCl particle filtration efficiency at 90 °C for 0. 75 h and 24 h, respectively.

In Figure 9, the initial respirator filtration efficiency and the corresponding changes upon exposure to DOP and NaCl particles at 80 °C for 0.75 h and 24 h, 90 °C for 0.75 h and 24 h were selected. When the dry heat treatment was applied for 24 h, the influence of DOP particles on the decrease in the filtration efficiency was greater than that of the NaCl particles. Moreover, the filtration efficiency decreased significantly with the increasing treatment time.

### 3.2. Microstructure

**Figure 10:**
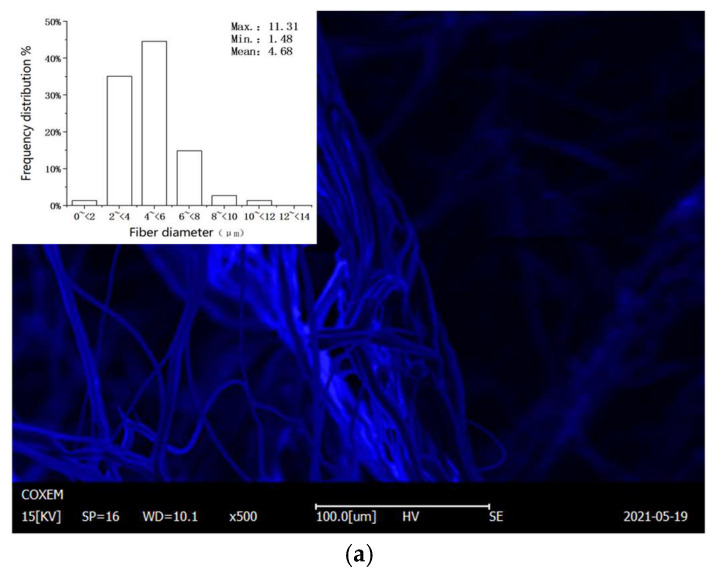

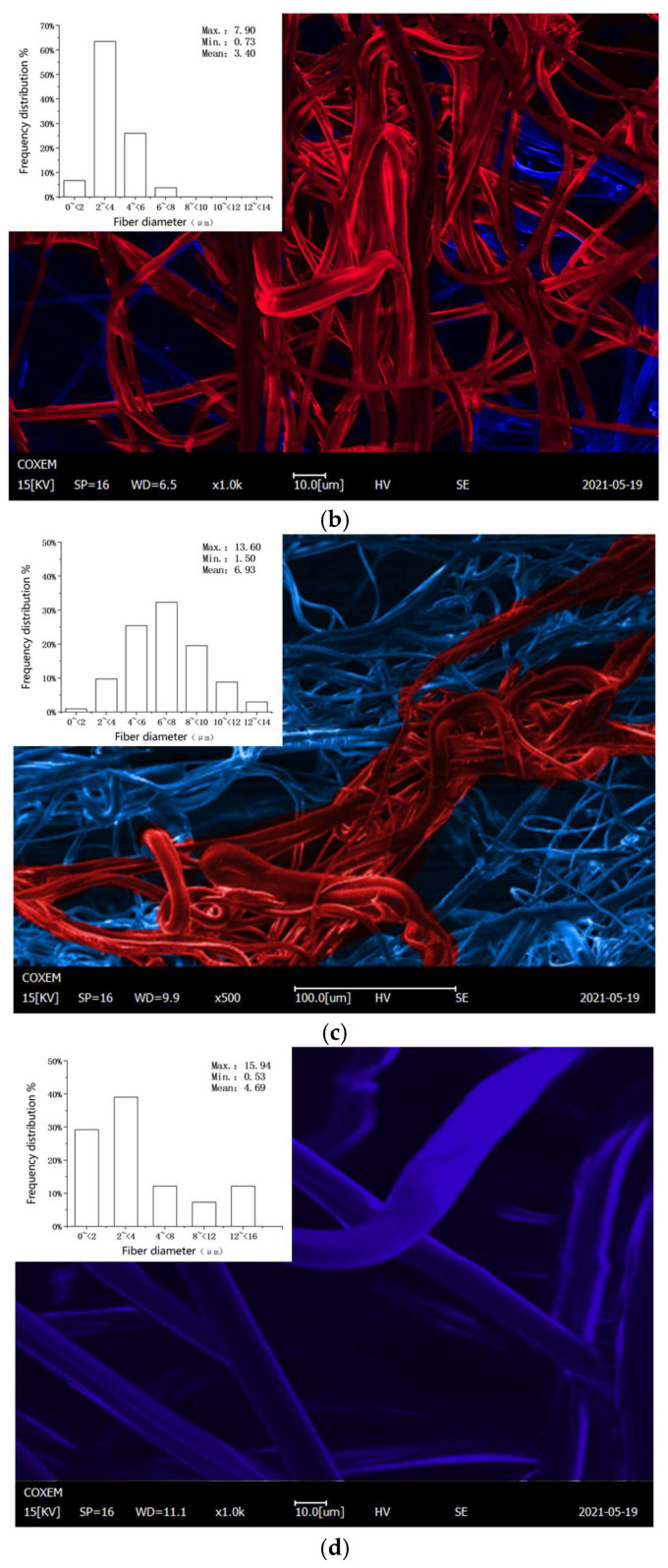

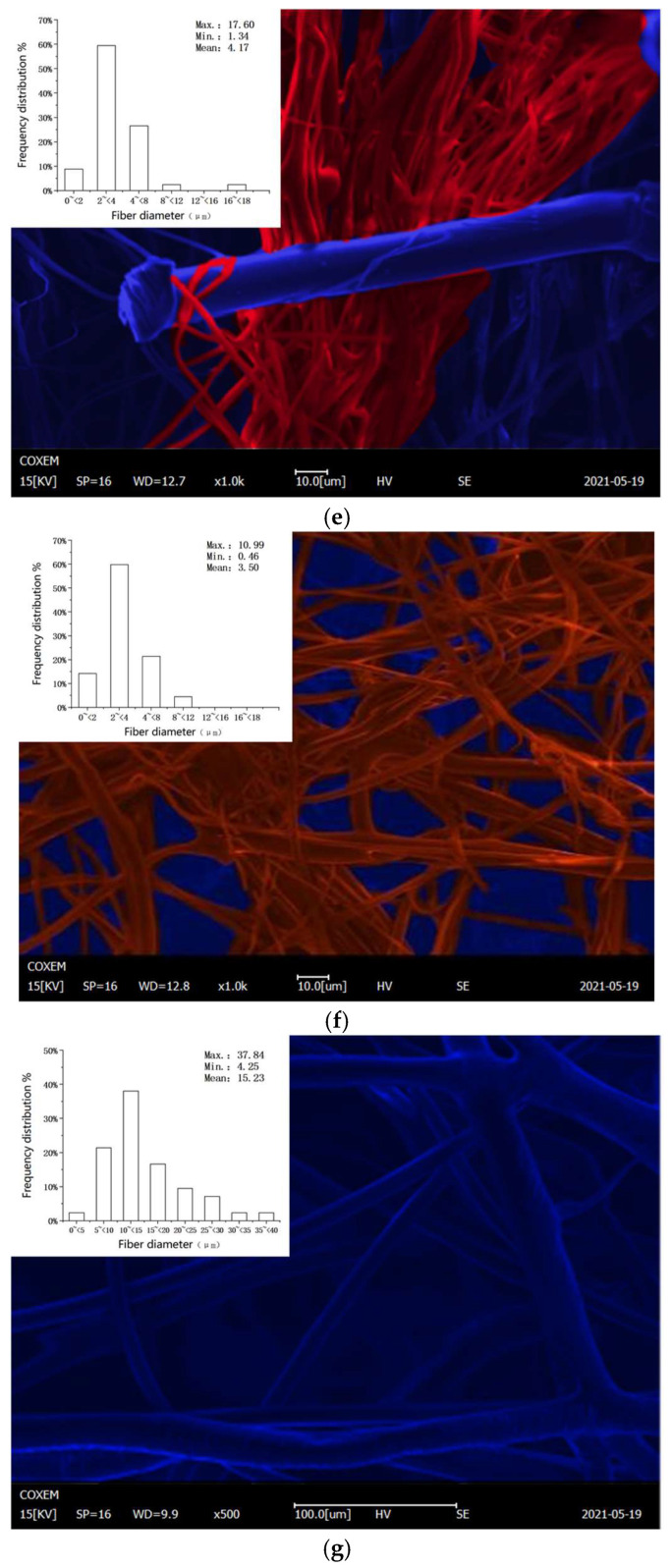

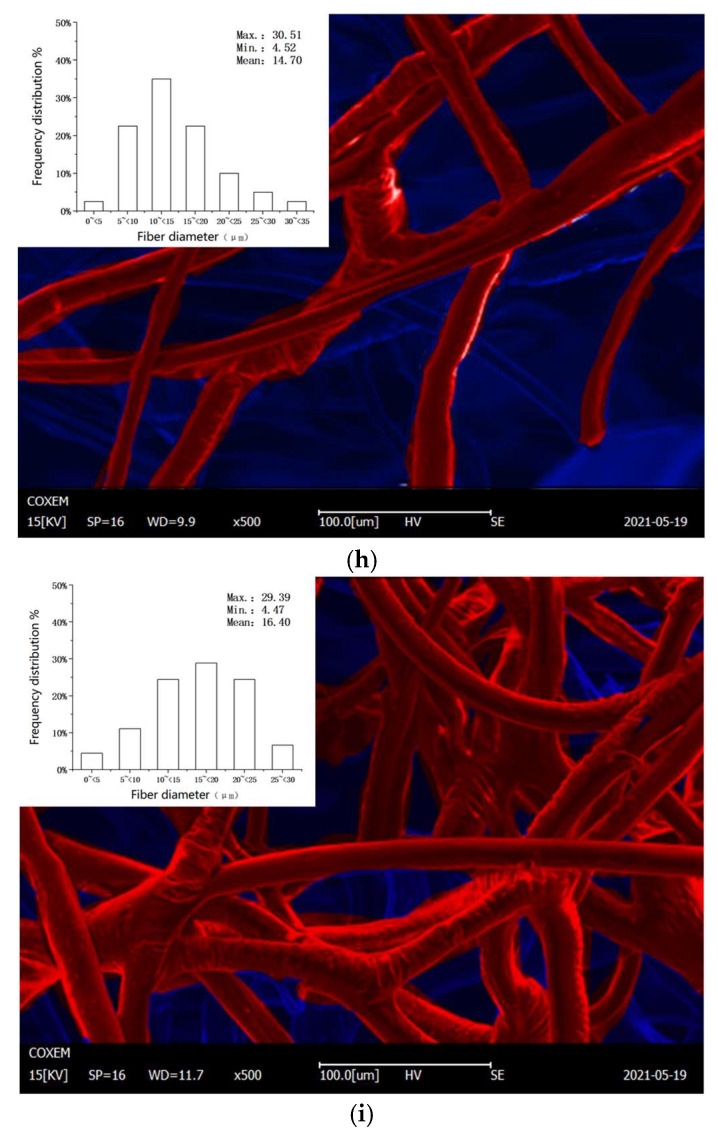
The SEM images of the (**a**) untreated 3M KN95 respirator, (**b**) 3M KN95 respirator at 80 °C for 24 h, (**c**) 3M KN95 respirator at 90 °C for 24 h, (**d**) untreated CM 8228-1 KN95 respirator, (**e**) CM 8228-1 KN95 respirator at 80 °C for 24 h, (**f**) CM 8228-1 KN95 respirator at 90 °C for 24 h, (**g**) untreated of CM 6002A KN95 respirator, (**h**) CM 6002A KN95 respirator at 80 °C for 24 h, and (**i**) CM 6002A KN95 respirator at 90 °C for 24 h. The upper left graphs are the fiber diameter distribution histogram.

The SEM images and fiber diameter distribution histogram of the melt-blown nonwoven fabrics of each respirator are shown in Figure 10. Figure 10a,d,g shows that the polypropylene filter of the respirator without dry heat treatment was intact and smooth. Figure 10b,c,e,f,h,i shows the polypropylene fibers with varying degrees of melting and agglomeration after heating at 80 °C and 90 °C for 24 h. The average diameter of the fibers of the melt-blown nonwoven fabric were in the order of 4.68 μm (3M, untreated), 3.40 μm (3M, 80 °C), 6.93 μm (3M, 90 °C); 4.69 μm (CM 8228-1, untreated), 4.17 μm (CM 8228-1, 80°), 3.50 μm (CM 8228-1, 90 °C); 15.23 μm (CM 6002A, untreated), 14.70 μm (CM 6002A, 80 °C), 16.4 μm (CM 6002A, 90 °C).

It can be seen from the SEM images that with the increase in temperature, the respirator fiber distribution inhomogeneity increases, the pore distribution range becomes wider, and the porosity becomes larger. The melting and agglomeration degree of polypropylene fiber heated at 90 °C was more serious than that at 80 °C. At the same temperature, the longer the heating time, the more obvious the filter melted and agglomerated. At the same time, the fiber diameter distribution graph showed that there was no obvious change in the fiber diameter with the increase in heating temperature for each type of respirator.

### 3.3. Model Building

Data were trained in an open-loop network with a serial-parallel structure, and the training effect is shown in Table 2. The regression (R) represents the fitting accuracy between the output and the target. The regression of all data was 0.87, and the regression of the training dataset, verification dataset, and test dataset was 0.86, 0.90, and 0.88, respectively, which were all close to 1. Thus, the trained network had relatively high accuracy. 

**Figure 11:**
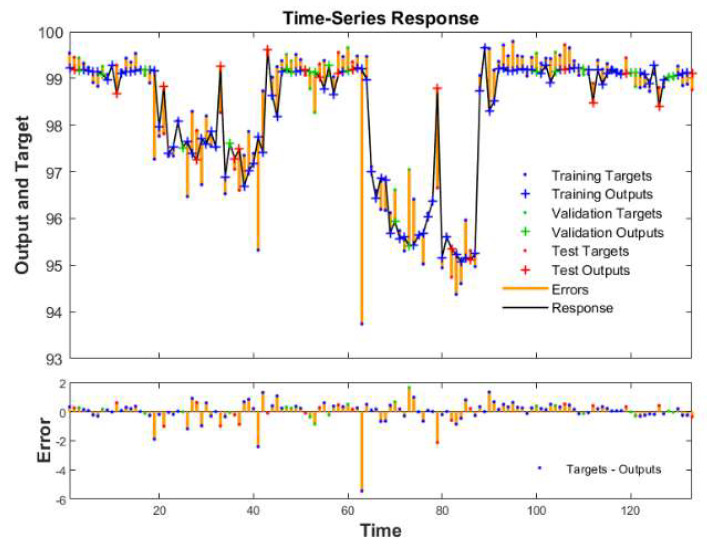
The time−series response.

We compared the filtering efficiency simulated by the network with the real filtering efficiency measured by the experiment, and the comparison results are shown in the Supplementary Materials. In Table S1 of the Supplementary Materials, the obtained data with a relative error less than 5% accounted for 96%, and the average relative error value was 0.5%. Figure 11 shows the fitting curve of the total data in the network. Outputs were the fitted values of the respirator filtration efficiency obtained by the neural network training, and targets were the actual values measured by the experiments. From the closed-loop in Figure 11, the trained network had a higher fitting accuracy for the peak value of the respirator filtration efficiency, while the fitting accuracy of the area with large variability in the filtration efficiency was slightly lower. The respirators’ filtration efficiencies calculated by the neural network were all 95% or above, accounting for 100% of all data. This shows that although the temperature and time of the heating treatment will affect the filtration efficiency, from 80 °C to 90 °C, the respirator filtration efficiency remained in a suitable range.

**Figure 12:**
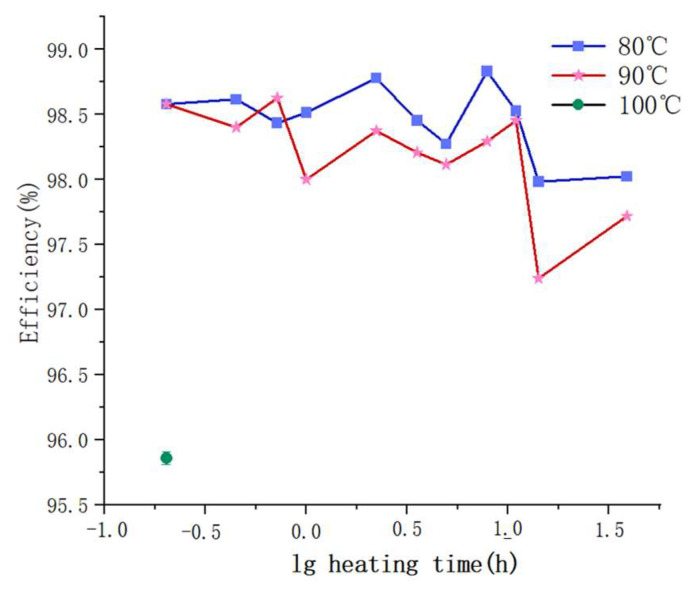
A comparison of the neural network prediction results and experimental results.

The prediction results of the neural network are shown in Figure 12. The predicted filtration efficiency obtained by the closed-loop structure of the neural network was 95.86% at 100 °C and 0.25, which was already approximate to the requirement of GB 2626-2019. The results showed that heat disinfection at 100 °C censured that the filtration efficiency of the respirator was close to the minimum filtration efficiency specified by the GB 2626-2019. If heated to a higher temperature or for a longer time, the filtration efficiency may continue to decrease.

## 4. Discussion

### 4.1. Variation of Filtration Efficiency

There is a nonlinear relationship between the filtration efficiency and heat treatment time and heating temperature. The heating temperature and time will affect the respirator filtration efficiency. The higher the temperature and longer the heating time, the more obvious the decline in filtration efficiency. In the SEM images, we found that the fiber diameter of the CM 6002A respirator was significantly larger than that of the other two brands. In addition to different fiber diameters, the biggest difference between the three types of respirators was the packaging. The CM 6002A KN95 respirator did not have an individual sealed wrapper as multiple respirators were sealed in one bag, which may lead to this type of respirator having the lowest initial filtration efficiency. The other two were completely sealed in individual wrappers. Therefore, the storage environment of the respirator may also affect the initial filtration efficiency.

### 4.2. Changes in Microstructure

The SEM images showed that the heating treatment will have an irreversible effect on the microstructure of the respirator filter. When the temperature increased, the filter fibers melted and agglomerated, resulting in an uneven spatial distribution of the microstructure of the filter [49]. The formation of larger voids led to an obvious decrease in the interception efficiency. This conclusion is consistent with Si et al., who reported on the changes in the microstructure of the polypropylene materials [50]. However, a recent study conducted by Liu did not find melting and agglutination of the microstructure after heating, but only observed an increase in the filter thickness after 110 °C treatment for 24 h [51]. The difference in the experimental results may be due to the difference in the production process of the polypropylene nonwoven fabric used and the quality of the products. At the same time, there were large differences in the fiber diameter of different brands of respirators.

### 4.3. Feasibility of Neural Network Method Application

We used the neural network as a mathematical tool to perform the regression analysis on the experimental data. The regression results further confirmed that extending the temperature and heating time will reduce the KN95 respirator filtration efficiency. We also successfully predicted the filtration efficiency of the respirator at 100 °C for 0.25 h. The use of this neural network is a tentative application of machine learning in the field of respiratory protection. This study verifies the feasibility of the neural network analysis method in analyzing the filtration efficiency of the respirators. At the onset of this study, we tried the BP neural network and NARX neural network to carry out a regression analysis on the experimental data. In the actual training process, the regression (R) of the BP neural network was lower than 50%. The regression (R) indicates that the BP neural network is not suitable for the data analysis. Compared with the BP neural network, the NARX neural network had increased certain sequence learning ability, which met our prediction needs. Therefore, the NARX neural network can realize effective prediction and an acceptable accuracy of filtering efficiency.

### 4.4. Feasibility of Dry Heat Disinfection

According to the experimental results and other authors’ research results [39,52], it is recommended that the heating disinfection temperature of the respirator should be between 70 °C and 90 °C, and that the time should be between 30 min and 2 h. Our conclusion is consistent with the optimum dry heat treatment temperature range obtained by Liao et al. [24]. Liao et al. examined the optimal number of treatment cycles for respirator disinfection. Based on this research, our team supplemented the effect of single dry heat treatment duration on the filtration efficiency. However, Oh et al. [37] proposed that the heating temperature of the respirator could reach 100 °C and the filtration efficiency could still be maintained above 95%. We hold the opinion that the main reason for this difference is the tested respirators. 3M respirators were used in the study of Oh et al., which showed significantly better initial filtration efficiencies than the CM respirators used in our study. To verify our point, we used the NARX neural network to perform the regression analysis on the experimental data of the 3M KN95 respirators used in our study. The regression results reached 82%, and the prediction results showed that the filtration efficiency reached 98.47% at 100 °C and 1 h. The predicted result is in agreement with the filtration efficiency result (above 95%, i.e., 97%) in the study of Oh et al. Therefore, the heating temperature and the time of the dry heat decontamination method are affected by the initial filtration efficiency of the respirator.

Due to the lack of conditions for the microbiological experiments, we did not conduct a microbial sampling study from the respirators before and after dry heat sterilization. However, compared with the treatment temperature (56 °C) and time (30 min) given by the China Diagnosis and Treatment Scheme for Novel Coronavirus Pneumonia (Trial Version 5) for inactivation of the virus [40], we reasonably increased the heating temperature and treatment time on the premise of ensuring the filtration efficiency. There are studies in the literature to prove that the inactivation efficiency of bacteria and viruses increases as the temperature rises [41,42,43]. Therefore, the recommendation of heating temperature and duration in our study is reasonably effective.

## 5. Conclusions

Three types of KN95 filtering facepiece respirators were treated at different temperatures and durations. The filtration efficiency of the respirator was tested at 80 °C and 90 °C for 15 min, 30 min, 45 min, 1 h, 2 h, 3 h, 4 h, 6 h, 8 h, 10 h, and 24 h. The filtration efficiency and microstructure of the KN95 respirator before and after heating were measured by a filter material detector and scanning electron microscope, respectively. The NARX neural network model was established to predict the filtration efficiency of the KN95 respirator.

The following conclusions are drawn:

(1) Heating temperature and duration will affect the respirator filtration efficiency. As the temperature and time increased, the decline in respirator filtration efficiency became more obvious.
(2) Through the SEM images, it is clear that excessive temperature led to the increase in the fiber distribution inhomogeneity and the pore distribution range, reducing the filtration efficiency of the respirator.
(3) The NARX neural network model exhibited an ideal fit. The model showed that the effect of dry heat treatment at 80 °C and 90 °C on the filtration efficiency of the respirator was limited when other factors were not considered. The respirator may have a surplus of filtration efficiency in the production process, therefore, the respirator may continue to be used after thermal disinfection at a certain temperature and duration. According to the predicted data of the neural network, it can be inferred that at 100 °C for 0.25 h, and above, the filtration efficiency of the respirator will no longer meet the requirement of GB 2626-2019.
(4) The initial filtration efficiencies of different respirators are inconsistent, which will lead to different reductions in the filtration efficiency caused by dry heating. According to our experimental results, the heating disinfection temperature can be between 70 °C and 90 °C, and the heating time can be between 30 min and 2 h.

## Figures and Tables

**Table 1:** The parameters of the particles.

Particle	Concentration	MMD *	NMD *
DOP	50–200 mg/m^3^	0.33 μm	0.20 μm
NaCl	12–20 mg/m^3^	0.26 μm	0.075 μm

* MMD—Mass median diameter; NMD—Number median diameter.

**Table 2:** Regression.

Training Regression (R)	Validation Regression (R)	Test Regression (R)	All Regression (R)
0.86	0.90	0.88	0.87

## Supplementary Materials

The following supporting information can be downloaded at: https://www.mdpi.com/article/10.3390/ijerph19127167/s1.
