# Supplementary material for: Dry Heat as a Potential Decontamination Method on the Filtration Efficiency of Filtering Facepiece Respirators
Source: Int J Environ Res Public Health. 2022 Jun 11;19(12):7167. doi: 10.3390/ijerph19127167 (PMC9223160; doi:10.3390/ijerph19127167)
Supplement: Supplementary file 1 [file ijerph-19-07167-s001.zip › ijerph-1707519-supplementary.pdf]

Supplementary material

Table S1 Neural Network Prediction Results and Experimental True Values

Targets	Outputs	Error	Ape	Targets	Outputs	Error	Ape	Targets	Outputs	Error	Ape
99.543	99.218	0.324	0.003	99.379	99.141	0.238	0.002	99.178	98.509	0.669	0.007
99.451	99.190	0.262	0.003	99.516	99.200	0.316	0.003	99.362	99.211	0.150	0.002
99.439	99.169	0.270	0.003	99.377	99.121	0.256	0.003	99.714	99.216	0.498	0.005
99.322	99.177	0.144	0.001	99.514	99.151	0.363	0.004	99.482	99.157	0.325	0.003
99.238	99.171	0.067	0.001	99.401	99.165	0.236	0.002	99.792	99.160	0.633	0.006
98.910	99.148	−0.238	0.002	99.073	99.178	−0.105	0.001	99.486	99.190	0.296	0.003
98.830	99.144	−0.314	0.003	98.776	99.150	−0.374	0.004	99.469	99.210	0.258	0.003
99.261	99.091	0.170	0.002	98.270	99.131	−0.861	0.009	99.049	99.185	−0.136	0.001
99.085	98.968	0.117	0.001	99.306	99.031	0.275	0.003	99.450	99.192	0.258	0.003
99.264	99.291	−0.027	0.000	99.383	98.779	0.603	0.006	99.537	99.152	0.385	0.004
99.282	98.677	0.605	0.006	99.054	99.290	−0.237	0.002	99.326	99.114	0.211	0.002
99.176	99.102	0.074	0.001	99.034	98.655	0.379	0.004	99.432	99.292	0.140	0.001
99.433	99.142	0.291	0.003	99.557	99.097	0.460	0.005	99.423	98.915	0.508	0.005
99.350	99.147	0.202	0.002	99.467	99.136	0.331	0.003	99.563	99.149	0.414	0.004
99.535	99.174	0.361	0.004	99.657	99.157	0.500	0.005	99.499	99.183	0.316	0.003
99.191	99.183	0.008	0.000	99.355	99.192	0.164	0.002	99.719	99.193	0.526	0.005
99.061	99.192	−0.131	0.001	99.481	99.216	0.265	0.003	99.655	99.206	0.449	0.005
98.903	99.180	−0.278	0.003	93.740	99.196	−5.455	0.058	99.377	99.223	0.154	0.002
97.270	99.161	−1.891	0.019	99.472	98.966	0.505	0.005	99.067	99.215	−0.148	0.001
97.763	97.964	−0.201	0.002	97.113	97.006	0.107	0.001	99.079	99.211	−0.132	0.001
97.815	98.829	−1.014	0.010	96.597	96.431	0.165	0.002	99.092	99.177	−0.085	0.001
97.343	97.401	−0.057	0.001	96.195	96.867	−0.672	0.007	98.898	98.485	0.412	0.004
97.338	97.531	−0.193	0.002	96.175	96.830	−0.655	0.007	99.389	99.193	0.197	0.002
98.100	98.081	0.019	0.000	96.122	95.693	0.429	0.004	99.195	98.862	0.333	0.003
97.503	97.519	−0.016	0.000	96.615	95.941	0.674	0.007	99.317	99.105	0.212	0.002
96.468	97.656	−1.188	0.012	95.742	95.568	0.174	0.002	99.221	99.180	0.041	0.000
98.293	97.385	0.908	0.009	95.307	95.611	−0.304	0.003	99.179	99.131	0.048	0.000
97.885	97.252	0.633	0.006	97.048	95.400	1.648	0.017	99.159	99.095	0.064	0.001
96.723	97.703	−0.979	0.010	96.413	95.427	0.987	0.010	99.441	99.112	0.329	0.003
98.198	97.594	0.603	0.006	95.623	95.643	−0.019	0.000	99.093	99.119	−0.026	0.000
97.545	97.858	−0.313	0.003	95.025	95.682	−0.657	0.007	98.825	99.127	−0.303	0.003
97.523	97.524	0.000	0.000	96.115	96.032	0.083	0.001	98.801	99.118	−0.317	0.003
98.270	99.258	−0.988	0.010	96.382	96.378	0.004	0.000	98.827	99.082	−0.255	0.003
96.528	96.884	−0.356	0.004	96.655	98.782	−2.127	0.022	98.722	98.890	−0.168	0.002
97.548	97.617	−0.069	0.001	94.940	95.158	−0.218	0.002	99.096	99.289	−0.193	0.002
97.055	97.286	−0.231	0.002	95.610	95.608	0.002	0.000	98.807	98.390	0.417	0.004
96.605	97.487	−0.882	0.009	94.740	95.342	−0.602	0.006	98.812	98.973	−0.161	0.002
97.352	96.678	0.674	0.007	94.373	95.239	−0.865	0.009	99.056	99.027	0.028	0.000
97.865	97.019	0.846	0.009	94.605	95.073	−0.468	0.005	99.000	99.044	−0.044	0.000
97.392	97.178	0.214	0.002	95.965	95.161	0.804	0.008	99.264	99.066	0.197	0.002

95.323	97.742	−2.419	0.025	95.312	95.105	0.206	0.002	98.847	99.105	−0.258	0.003
98.731	97.417	1.314	0.013	94.970	95.244	−0.274	0.003	98.874	99.125	−0.250	0.003
99.507	99.616	−0.109	0.001	99.063	98.731	0.332	0.003	98.755	99.111	−0.356	0.004
99.026	98.634	0.392	0.004	99.655	99.661	−0.005	0.000	99.639	98.299	1.340	0.013
99.253	98.177	1.076	0.011								
